# Editorial: The upcoming complications of COVID-19 on recovered patients: molecular mechanisms and therapeutic opportunities, volume II

**DOI:** 10.3389/fmolb.2023.1263356

**Published:** 2023-08-15

**Authors:** Seyed Hassan Seradj, William C. Cho, Zohreh Amoozgar, Rana Jahanban-Esfahlan

**Affiliations:** ^1^ Department of Medicinal Chemistry, School of Pharmacy, Shiraz University of Medical Sciences, Shiraz, Iran; ^2^ Department of Clinical Oncology, Queen Elizabeth Hospital, Kowloon, Hong Kong SAR, China; ^3^ Edwin L. Steele Laboratories, Department of Radiation Oncology, Massachusetts General Hospital and Harvard Medical School, Boston, MA, United States; ^4^ Department of Medical Biotechnology, Faculty of Advanced Medical Sciences, Tabriz University of Medical Sciences, Tabriz, Iran

**Keywords:** COVID-19, molecular mechanisms, long-term effects, recovered patients, therapeutic opportunities

On 5 May 2023, after more than 3 years of battle against COVID-19, the World Health Organization (WHO) officially announced the end of the global Public Health Emergency for COVID-19 (Wise, 2023). However, global issues brought on by the COVID-19 pandemic require ongoing investigation to improve our knowledge of the virus, its effects on individuals, and prospective treatments.

Our Research Topic addresses two emerging challenges encountered by COVID-19 patients: the re-detectable positive (RP) phenomenon and the long-term effects commonly referred to as long COVID. Furthermore, first-time population-based pharmacovigilance analysis for autoimmune hepatitis following COVID-19 vaccination was reported, and prospective therapeutic targets with a focus on PI3K/Akt/Nrf2 mediated cellular signaling pathway were discussed. We aim to provide valuable insights into the continuing challenges arising from this global pandemic.

Peng et al. studied SARS-CoV-2 recurrence in individuals who have previously recovered from COVID-19 infection. Blood cell transcriptome sequencing reveals slight genomic discrepancies. In this study, the most distinguishing factors between re-detectable positive (RP) and non-re-detectable positive (NRP) patients were sex, IgG and IgM titers in the blood. The number of female RP patients was substantially high (67.8%), and the titers of IgG and IgM antibodies were also significantly raised. Differentially expressed genes (DEGs) were mainly involved in antigen recognition, inflammatory modulation, and immune responses. Co-expression networks from two mutually verified RP datasets were constructed, and 59 overlapping indicator genes were highly correlated with RP results. Therefore, along with long-term isolation and multiple nucleic acid tests, transcriptional sequencing can be a useful complementary strategy for the early and reliable diagnosis of RP patients.

Kenny et al. highlighted the multifaceted nature of long COVID, encompassing respiratory, cardiovascular, neurological, and immunological manifestations. A thorough investigation is conducted in this all-encompassing review on the clinical characteristics, potential pathogenesis, and therapeutic targets for long COVID and they suggested pathogenic pathways involve immune dysfunction, sustained viral reservoirs, and tissue damage. Individuals with long COVID-19 have been observed with persistent T cell activation and alterations in immune populations, such as a decrease in the number of naïve B cells. Increased innate cell activation, altered cytokine levels, tissue viral persistence, autoimmunity, dysbiosis, dysautonomia, and endothelial dysfunction have also been linked to the development of long COVID-19.

Prompt reporting of potential adverse events and implementation of robust pharmacovigilance systems are critical in the ongoing global vaccination campaigns. The administration of COVID-19 vaccinations has led to a rapid emergence of anecdotal reports on autoimmune hepatitis (AIH) following vaccination (Zheng et al., 2022). Reports have led to concerns regarding the potential rise in the risk of AIH due to COVID-19 vaccination, thereby resulting in vaccine hesitancy. To date, no population-based study has been conducted to address this apprehension. Chen et al. reported a total of 53 cases of autoimmune hepatitis (AIH) which were identified between 11 December 2020 and 15 March 2022 collected from the Centers for Disease Control (CDC) COVID Data Tracker and the Vaccines Adverse Event Reporting System (VAERS) after administration of COVID-19 vaccine. The overall rate of AIH that was reported in relation to COVID-19 vaccination was 0.21 (95% CI 0.16–0.27) per million individuals. The findings reveal that the AIH cases were lower than expected suggesting that post-vaccination autoimmune hepatitis cases may not pose a safety concern for COVID-19 vaccination.

Lekshmi et al. reviewed the molecular mechanisms underlying the SARS-CoV-2 infection process, identifying potential therapeutic targets associated with the PI3K/Akt/Nrf2 cellular signaling pathway. The activation of Nrf2 has been demonstrated to alleviate the inflammatory response and oxidative stress induced by SARS-CoV-2 infection. The modulation of this pathway holds significant potential for the development of effective therapeutic interventions against COVID-19.

In summary, this Research Topic offer valuable insights into various aspects of COVID-19 such as recurrence, long COVID, vaccine-related adverse effects and potential therapeutic targets. These studies significantly contribute to the existing knowledge base as the global scientific community continues to deepen its understanding of this virus. Translating these research findings into clinical practice empowers healthcare professionals to optimize strategies for managing COVID-19 and its long-term implications. Hence, continuous research efforts remain crucial in our collective fight against this unprecedented global health crisis.
